# Do Surface Charges on Polymeric Filters and Airborne Particles Control the Removal of Nanoscale Aerosols by Polymeric Facial Masks?

**DOI:** 10.3390/toxics12010003

**Published:** 2023-12-19

**Authors:** Zhaobo Zhang, Mahmut S. Ersan, Paul Westerhoff, Pierre Herckes

**Affiliations:** 1 School of Molecular Sciences, Arizona State University, Tempe, AZ 85297-1604, USA; zzhan223@asu.edu; 2 NSF Nanosystems Engineering Research Center for Nanotechnology-Enabled Water Treatment, School of Sustainable Engineering and the Built Environment, Ira A. Fulton Schools of Engineering, Arizona State University, Tempe, AZ 85287-3005, USA; mahmut.ersan@und.edu (M.S.E.); p.westerhoff@asu.edu (P.W.); 3 Department of Civil Engineering, University of North Dakota, Grand Forks, ND 58202-8115, USA

**Keywords:** latex particle, filtration efficiency, surface potential, surface charge

## Abstract

The emergence of facial masks as a critical health intervention to prevent the spread of airborne disease and protect from occupational nanomaterial exposure highlights the need for fundamental insights into the interaction of nanoparticles (<200 nm) with modern polymeric mask filter materials. While most research focuses on the filtration efficiency of airborne particles by facial masks based on pore sizes, pressure drop, or humidity, only a few studies focus on the importance of aerosol surface charge versus filter surface charge and their role in the net particle filtration efficiency of mask filters. In this study, experiments were conducted to assess mask filter filtration efficiency using positively and negatively charged polystyrene particles (150 nm) as challenge aerosols at varying humidity levels. Commercial masks with surface potential (Ψ_f_) in the range of −10 V to −800 V were measured by an electrostatic voltmeter and used for testing. Results show that the mask filtration efficiency is highly dependent on the mask surface potential as well as the charge on the challenge aerosol, ranging from 60% to 98%. Eliminating the surface charge results in a maximum 43% decrease in filtration efficiency, emphasizing the importance of electrostatic charge interactions during the particle capture process. Moreover, increased humidity can decrease the surface charge on filters, thereby decreasing the mask filtration efficiency. The knowledge gained from this study provides insight into the critical role of electrostatic attraction in nanoparticle capture mechanisms and benefits future occupational and environmental health studies.

## 1. Introduction

In the past few years, the severe acute respiratory syndrome coronavirus 2 (SARS-CoV-2) pandemic has highlighted the importance of transmission of aerosols [1,2,3,4,5,6]. This has led to public discussions on the usefulness of masks and an emergence of evaluation studies on the efficiency of commonly used masks to capture aerosols [7,8,9,10]. While these studies have provided valuable information on mask filtration efficiency, only a limited number of them have addressed mechanistic aspects. As a result, there is still a lack of understanding of the actual aerosol capture mechanisms, particularly towards airborne nanoparticles. 

Airborne nanoparticles are widely present across environmental settings. They can be generated by both natural sources, such as forest fires [11] or biogenic nucleation events [12,13], and anthropogenic sources, such as biomass burning [14] and industrial manufacturing activities [15], as well as vehicle emissions [16]. Even aerosols from sources typically associated with larger particles, such as volcanic eruptions [17] or marine aerosols, do contain nanosized particles [18,19]. In contrast to larger aerosols, such as PM_10_ (particulate matter with a diameter of 10 µm or less) or respiratory droplets (the majority are exhaled aerosols <5 µm in diameter), which settle rapidly [20,21,22], airborne nanoparticles have a longer residence time and can travel longer distances, increasing their spatial reach [23]. Exposure to airborne nanoparticles has become a growing concern due to studies showing that they can be inhaled and potentially penetrate vascular walls, leading to translocation to different organs [24,25]. Comprehending the filtration efficiency and mechanism of prevalent masks in relation to airborne nanoparticles is a growing area of need for public and occupational health. 

Filtering facepiece respirators (FFR), such as N95 masks, show over 95% filtration efficiency towards airborne particles under conditions defined by the National Institute for Occupational Safety and Health (NIOSH) [26]. N95 and N95 equivalent-level masks are usually made of a four-layer structure. Figure S1a shows a common design where a non-woven fabric (e.g., polypropylene) outer layer prevents liquid droplets and blocks bacteria, germs, or visible dust. The middle layer is the filter layer, which is made of an electrostatically charged microfiber filter (melt-blown non-woven fabric). A hard plastic supporting shell (third layer) is typically attached to the filter layer to prevent collapsing. A secondary inner layer includes a hydrophobic, non-woven fabric layer that minimizes the breathing-out moisture that can damage respirators and also blocks droplet spreading from exhalation [27]. Surgical masks (Figure S1b) are another type of commonly used mask during pandemics or regular industrial manufacturing processes that typically have a three-layer structure. Outer and inner layers are made of non-woven hydrophobic fabric to prevent inhalation/exhalation of liquid droplets. The middle layer is used to capture contaminant particles and typically includes a melt-blown fabric or Spun-Bond fabric layer [28,29,30]. There is no specific requirement for the filtration efficiency of surgical masks. Based on past studies, Morais et al. have tested nine different surgical masks using polydisperse sodium chloride challenge aerosol particles in the size range of 60–300 nm, and they observed varying filtration efficiency, ranging from 75% to 88% [31]. These findings highlight inconsistent performance among surgical masks. Furthermore, Rengasamy et al. assessed five surgical masks using 100 nm polystyrene latex (PSL) particles and room air particles. They reported higher filtration efficiency (>98%) for PSL particles compared to room air particles (ranging from 83.4% to 92%) [32]. These studies demonstrate that both the type of challenge aerosol and the specific surgical mask influence filtration efficiency. However, the underlying reasons for these differences remain unknown. 

Aerosol capture mechanisms by filters follow mostly the five basic processes: gravity sedimentation, inertial impaction, interception, electrostatic attraction, and diffusion [33,34], although additional mechanisms, such as dielectrophoretic filtration, have been proposed too [35]. Gravity sedimentation, impaction, and interception primarily control the capture of larger aerosols over >1 µm. For sub-micrometer-range aerosols (<1 µm), interception and impaction decline, and electrostatic attraction tend to be the dominant mechanisms for achieving efficient particle capture. Diffusion becomes an important mechanism for capturing particles smaller than 0.2 µm. Within this size range, particles are subject to Brownian motion and move randomly, caused by the interactions with fluid molecules, increasing the likelihood of collisions with filter material [30,36]. The existence of charge in FFR filters can improve the filtration efficiency of FFR significantly through electrostatic interactions. Some studies have reported that when treated with isopropanol to remove surface charge, the FFR efficiency drastically decreases [37,38,39]. Previous studies have reported that surgical mask melt-blown filter layers can also have a surface charge, although their surface charge strength and distribution are not discussed in the literature [40,41]. Based on the aerosol capture mechanisms, whether it is the official NIOSH method or past research [26,31,32], mask filtration efficiency testing tends to use the most penetrating aerosol particles (<0.3 µm) as the test aerosol. Compared to these small particles, larger particles or even droplets common in exhaled air are more easily captured through mechanisms such as inertial impaction and interception.

There are few studies that have systemically evaluated the effects of varying charged particles and their interaction with masks of differing surface charge, as most mask materials are commercially produced by proprietary processes and are difficult to comprehensively study [32,42,43]. In this study, we aim to address these challenges by using monodisperse 150 nm functionalized (amine, carboxylic acid, sulfate) PSL particles and challenge masks with different surface potentials. We tested a range of filters through an experimental matrix with humidity control to determine (i) the relationship between filtration efficiency and mask surface potential, (ii) the filtration efficiency of masks against differently functionalized PSL particles with varying surface charge states, and (iii) the aerosol filtration efficiency of masks after they lose their surface charge. Finally, the impact of relative humidity on the test results is also considered. 

## 2. Materials and Methods

### 2.1. Materials

In this study, 150 nm non-functionalized PSL nanospheres were obtained from Thermo Scientific Inc. (Waltham, MA, USA); 150 nm carboxylic acid-functionalized PSL particles (carboxylic acid-PSL) were ordered from Bangs Laboratories Inc. (Fishers, IN, USA). The 150 nm amine-functionalized PSL particles (amine-PSL) were ordered from Creative Diagnostics Inc. (Shirley, NY, USA). 2-propanol (Optima grade) was obtained from Fisher Scientific Inc. (Hampton, NH, USA). Potassium chloride (KCl) was obtained from Sigma-Aldrich Inc. (St. Louis, MO, USA). Hydrochloric acid (HCl, ACS grade) was obtained from Sigma-Aldrich Inc. (St. Louis, MO, USA). Sodium hydroxide (NaOH) was obtained from Mallinckrodt Inc. (Saint Louis, MO, USA). The masks used in this study were all purchased and commercially available.

### 2.2. Filtration Efficiency Test 

**Figure 1:**
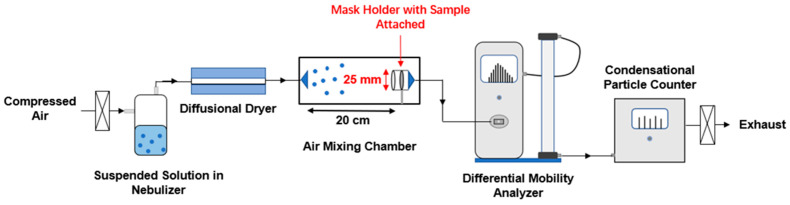
Schematic diagram of mask filtration efficiency testing setup.

This study evaluated the filtration efficiency of eleven commercial masks, including four N95 masks and seven surgical masks. Figure 1 illustrates the setup for testing the filtration efficiency of masks. A commercial medical nebulizer (Power Nebulizer Ultra, Drive Medical, Port Washington, NY) was used to generate a consistent aerosol flow from an aqueous particle suspension [38,44,45], which contained one of the following particles: 150 nm non-functionalized PSL nanospheres, 150 nm carboxylic acid-PSL, or 150 nm amine-PSL. The nebulized aerosol flow was then conditioned to a set relative humidity (RH), e.g., 40% and 80%, using a diffusional dryer (TSI 3062, Shoreview, MN, USA). Sections of the mask were cut using a 25 mm internal diameter punch and attached to an open-face filter holder (SKC 225-3-25LF, Eighty Four, PA, USA). Mask samples inside could be flipped for the dual-direction testing to ensure consistent performance during both inhalation and exhalation. The airflow inside the system was maintained at 1.5 L/min, corresponding to a face velocity of 5.1 cm/s across the filter. Particles in the absence (control) or presence of masks were quantified in situ using a Scanning Mobility Particle Sizer (SMPS) consisting of an Electrostatic Classifier (model 3082, TSI Inc., Shoreview, MN, USA), a soft X-ray charge neutralizer (model 3088, TSI Inc., Shoreview, MN, USA), a Differential Mobility Analyzer (DMA, model 3081A; TSI Inc., Shoreview, MN, USA), and a Condensational Particle Counter (CPC, model 3752; TSI Inc., Shoreview, MN, USA). In this study, the SMPS was used to measure the size-resolved (20–220 nm) particle number concentration with the assistance of TSI AIM software [46]. The sheath flow inside DMA was maintained at 15 L/min. Additional parameters, including reference gas viscosity, gas temperature, and pressure, are provided in Table S1. There was no overpressure or underpressure, and no overheating or underheating was observed during operation processes.

The mask filtration efficiency was calculated based on the following equation, using the interval of maximum peak around 150 nm, which was observed on the spectrum from the SMPS:

$$Filtration\;efficiency=(1-\frac{C_{filter}}{C_{control}})\times 100\%$$

where *C_filter_* and *C_control_* represent the average total particle number concentration for the sample and control, respectively. The particle number concentration of *C_filter_* was measured when the mask material was inserted and was quantified through triplicate measurements with the SMPS. The difference in integrated particle number concentration between intervals of maximum peak was consistently within 5%. The particle number concentration of *C_control_* was measured without the presence of mask material. In contrast to the *C_filter_* measurement, *C_control_* was measured six times (three measurements before and after mask tests, respectively) to ensure the consistency of the blank level before and after the mask tests [30,31].

### 2.3. Mask Surface Potential

**Figure 2:**
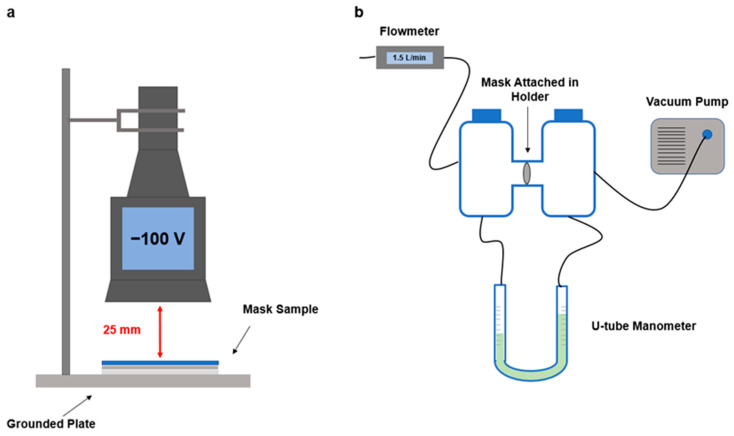
Schematic diagram of (**a**) surface potential measurement setup and (**b**) pressure drop measurement setup.

Figure 2a shows the experimental setup used to measure the mask surface potential (Ψ_f_) using an electrostatic voltmeter (Keyence Inc., Itasca, IL, USA) [43,47,48]. Following the system manual, the detecting surface (0.56 cm^2^) was placed 25 mm above a grounded plate. To avoid heterogeneity of surface potential distribution on the sample surface, the surface potential was measured at 10 different random positions on each sample and then averaged. Each measurement was repeated three times to ensure replicability [49].

To eliminate surface charge, mask samples were soaked in 75% 2-propanol for 10 min and dried overnight [38]. After drying, the surface potential of each mask was measured and was found to be around 0 V, regardless of mask type (e.g., N95 or surgical mask).

### 2.4. Particle Surface Charge

Zeta potentials (Ψ_zeta_) for particles dispersed in a 1 mM KCl solution were measured using a Zeta potential analyzer (Nanobrook series; Brookhaven Inc., Holtsville, NY, USA) [50]. Particle number concentrations were adjusted to achieve an instrument reading not exceeding 1000 kcps in order to follow the instrument instructions [51]. The solution pH (Table S2) was measured using a pH meter (model SB80PD, VWR Inc., Radnor, PA, USA) and adjusted, if necessary, using 10 mM NaOH and 10 mM HCl to match the specific pH (pH = 6.06 for carboxylic acid-PSL, pH = 6.23 for PSL, pH = 9.12 for amine-PSL) level of challenge particle suspension which used for filtration efficiency tests. The average Zeta potential results, along with standard deviations, are presented in Table S2 as Ψ_zeta_ based upon triplicate measurements. 

The particle surface charge in the gas phase was evaluated by operating the SMPS without turning on the soft X-ray charge neutralizer, which prevents the neutralizer from providing a bipolar equilibrium charge state on aerosols. Then, the charged particles could be selected by DMA based on size distribution, pass through the analyzer, and be detected by CPC. However, the neutral particles were not affected by the electric field inside DMA due to lack of surface charge. These neutral particles were directly removed by the sheath flow and were unable to reach the exit of the DMA to be detected by the CPC [52]. Additionally, for particles with different polarities, comprehensive characterization required the use of a dual-polarity mode in the DMA. However, this was not implemented in this study due to instrumentation limitations. Consequently, only the negatively charged SMPS spectrum has been characterized in this study.

### 2.5. Pressure Drop Measurement across Filters

The pressure drop (∆P) was measured by a U-tube manometer (model 1211; Dwyer Inc., Michigan City, IN, USA), which connected on both sides to the mask material [31]. The mask was attached to a filter holder with a 25 mm diameter circular exposure area (Figure 2b). The airflow passed through the mask material had a consistent flow rate of 1.5 L/min (face velocity of 5.1 cm/s), which was measured by a mass flowmeter (mode 4100; TSI Inc., Shoreview, MN, USA). The pressure drop results are shown in Table 1 in units of mm H_2_O/cm^2^.

## 3. Results and Discussion

### 3.1. Mask Characterization Results: Surface Potential, Filtration Efficiency and Pressure Drop

Table 1 displays key measurements of N95 masks and surgical masks, including surface potentials, filtration efficiency, and pressure drop. The surface potential of each mask ranged from near neutral (−10 ± 5 V) to highly charged (−800 ± 108 V), with standard deviations indicating variability across filter materials. N95 masks exhibited higher surface charge (−230 ± 30 to −800 ± 108 V) compared to surgical masks (−10 ± 5 to −160 ± 18 V). Overall, the masks used in our study showed slightly lower net surface potential (disregard polarity) than those reported in previous studies on FFRs (~1100 V) and surgical masks (~250 V) [27], while a different study reported surface potentials from −375 V to −484 V for surgical masks [53]. The variability observed between studies and within our study indicates that the polarity and strength of the surface potential vary depending on the manufacturer, but overall, FFRs tend to have higher surface potential than surgical masks. Additionally, the surface potential can be influenced by the age of the mask, as surface charge may gradually dissipate through neutralization, leading to a decrease in surface potential. At the consumer level, no information was available on the age of the masks tested. In this study, the masks used were ordered before testing, and no long-term storage changes in surface potential were observed (Table S3).

The filtration efficiencies of each mask were evaluated by challenging them with 150 nm aerosolized PSL particles, as shown in Table 1. The N95 masks exhibited a high filtration efficiency, with over 99% of 150 nm PSL particles being captured in the experiment. There was no significant difference (*p* > 0.05) in filtration efficiency observed between different N95 mask samples as a group based on a two-tailed unpaired t-test. In contrast, the average filtration efficiency of surgical masks varied between samples, ranging from 82.7 ± 1.4% to 98.1 ± 0.3%. Overall, the surgical masks tested showed high variability, and there were significant differences (*p* < 0.05) in filtration efficiencies within this group as a whole. Additionally, a dual-direction experiment was also conducted, which demonstrated that the filtration efficiency of masks in both inhalation and exhalation directions showed no significant difference (*p* > 0.05). The results of this experiment are shown in Figure S2.

Furthermore, it is important to note that we intentionally refrained from neutralizing the challenge aerosol before conducting the filtration efficiency test. Consequently, the extreme charge state in freshly generated aerosols may lead to an overestimation of filtration efficiency when contrasted with the Boltzmann distribution of charge states typically found in atmospheric aerosols [8]. In the study, our primary goal was to investigate the influence of both mask and challenge aerosol surface charge on filtration efficiency, which prevents us from pre-neutralizing the challenge aerosols prior to filtration efficiency tests.

In our study, N95 masks demonstrated a high pressure drop, ranging from 6.5 to 9 mm H_2_O (63.7–88.3 Pa), consistent with other studies (60–90 Pa) [27,38]. For surgical masks, the observed pressure drop varied across a wider range, from 3 to 8 mm H_2_O (29.4–78.5 Pa). According to the filtration efficiency values presented in Table 1, it is evident that pressure drop is not the primary determinant of surgical mask filtration efficiency. Notably, Surgical Mask C (97.7 ± 0.4%) and Surgical Mask F (90.9 ± 1.8%) exhibit the same pressure drop (4.5 mmH_2_O/cm^2^), yet they demonstrate significant differences in filtration efficiency. From a usage perspective, the quality factor can be an indicator of mask quality and can be calculated based on filtration efficiency and pressure drop, as shown in Table S4. A higher quality factor means the mask has higher filtration efficiency and lower pressure drop [8,31]. The results suggest that the quality factor is independent of the type of mask.

We used scanning electron microscopy to image each layer of the masks, as shown in Figure S3. Notably, across both N95 and surgical masks, we observed that Layer 1 and Layer 3 present a similar fiber diameter of approximately 20 µm, while Layer 2 shows a smaller fiber diameter of approximately 3 µm. These consistent measurements among the masks indicate that there is no difference in fiber diameter, thus avoiding any potential influence on filtration efficiency due to variations in this parameter.

### 3.2. Particle Surface Charge Characterization Results

The results of Zeta potential (Ψ_p_) measurements for each challenge aerosol suspension are shown in Table S2; the measurements were performed at the pH level at which the suspensions were aerosolized. The results showed that the PSL particles contained a slightly negative charge (Ψ_p_ = −22 ± 2 mV) due to the presence of sulfate groups on the terminal of the polymer chain but were not considered functionalized PSL particles. The two functionalized PSL particles showed different surface charges due to their functionalization. Carboxylic acid functionalization led to a higher negative surface charge (Ψ_p_ = −60 ± 3 mV), while amine functionalization resulted in a positive surface charge (Ψ_p_ = 21 ± 1 mV). However, it is important to note that the Zeta potential of each PSL particle in suspension does not necessarily represent the surface charge when they are nebulized.

Aerosolized particles have an unknown surface charge distribution and increase the difficulty of particle size distribution analysis. To address this, a bipolar neutralizer was employed in the SMPS system. This neutralizer continuously generates positive and negative ions inside a chamber, allowing the aerosol to reach charge equilibrium states based on Boltzmann’s Law, as described in past studies by Fuchs [54] and Wiedensohler [55]. Through this neutralization process, the inlet aerosol with an unknown surface charge distribution is transformed into an outlet aerosol with a known distribution of surface charge states, primarily consisting of low surface charges (typically one or two elemental charges on the surface) [52,56]. Next, the aerosol flow output from the bipolar neutralizer enters the DMA, where particles are selected based on their electrical mobility diameter. Particles with a single elementary charge have an electrical mobility diameter close to their actual physical diameter, while particles with higher charges have smaller electrical mobility diameters compared to their actual physical diameter. Particles with no surface charge are removed in the DMA.

**Figure 3:**
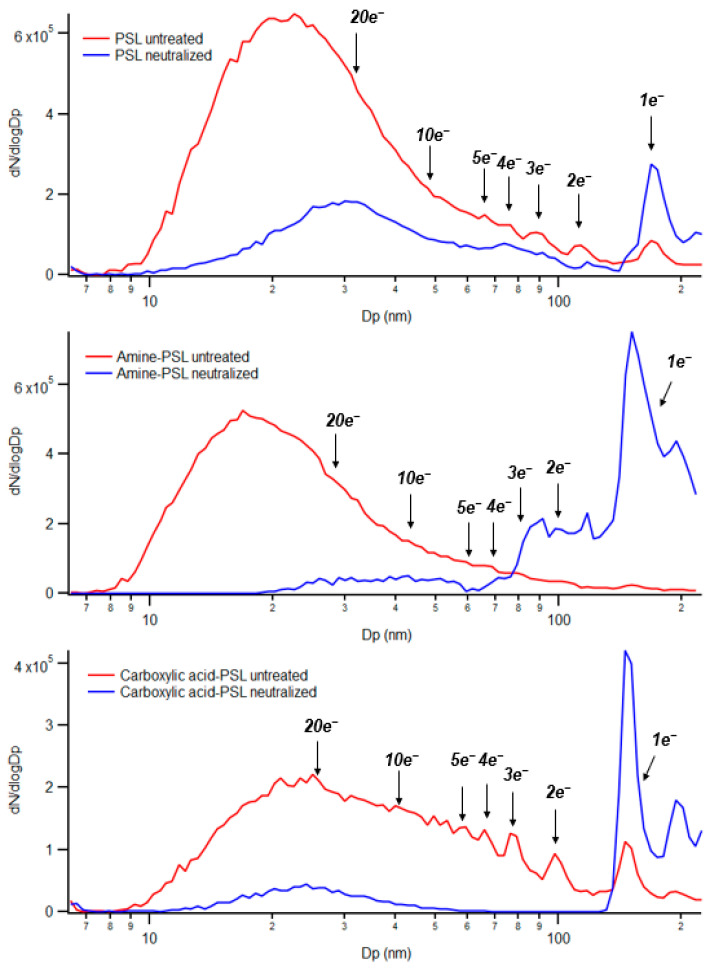
Negatively charged SMPS spectra of three different PSL particles (unfunctionalized PSL, amine-PSL, carboxylic acid-PSL). The blue line represents particle number concentration measured after surface charge neutralization, while the red line represents particle number concentration measured without the surface charge neutralization step. The spectra are labeled with the number of electrons, indicating the peak positions corresponding to the electrical mobility diameter.

To verify the surface charge and its state of challenge aerosol after nebulization, we monitored the aerosols using SMPS without passing through the bipolar neutralizer. This setup prevented the aerosolized particles from being neutralized inside the SMPS, allowing us to characterize the surface charge after nebulization. The characterization included determining whether particles retained their surface charge and the number of elementary charges on each particle. Figure 3 shows the negatively charged particle spectra measured by SMPS, encompassing all three PSL particles with and without neutralization processes. The number of elementary charges and their corresponding peak positions were labeled in the spectra. The number of electrons and their corresponding electrical mobility diameter are presented in Table S5, following the calculation method described by Laengert et al. [52].

Based on Figure 3, all three PSL particles exhibit a prominent peak around 150 nm when the aerosol has been neutralized. This suggests that after neutralization, most particles carry only a single elementary charge, the particle size aligning with the expectation (150 nm). However, when each PSL particle remains untreated without surface charge neutralization, it shows distinct phenomena. The unfunctionalized PSL and carboxylic acid-PSL particles still show a distinct peak around 150 nm, which indicates that the particle possessed a single negative elementary charge, so the particle had an electrical mobility diameter close to its original size property (150 nm). In contrast, the amine-PSL particles show no distinct peak across the testing range (~150 nm), indicating that they may not be charged or carry the opposite (+) charge. Unfortunately, our SMPS instrument does not support polarity flip; hence, an opposite polarity experiment was not possible in this study. The broad peak observed in the range of 10–50 nm for amine-PSL particles is more likely attributed to other species, such as agglomerated surfactants, rather than highly charged particles (>10 e). This is supported by past studies [52,55], which indicate that particles with higher number concentrations are more likely to have lower charged particles rather than highly charged ones. This is also evident in the neutralized spectrum of unfunctionalized PSL, where some peaks are present in this range (10–50 nm). Overall, amine-PSL particles tend to be either neutral or positively charged, in contrast to unfunctionalized PSL and carboxylic acid-PSL particles that maintain a negative surface charge after nebulization.

### 3.3. Impact of Mask Surface Potential (Ψ_f_) on Filtration Efficiency of PSL Particles

**Figure 4:**
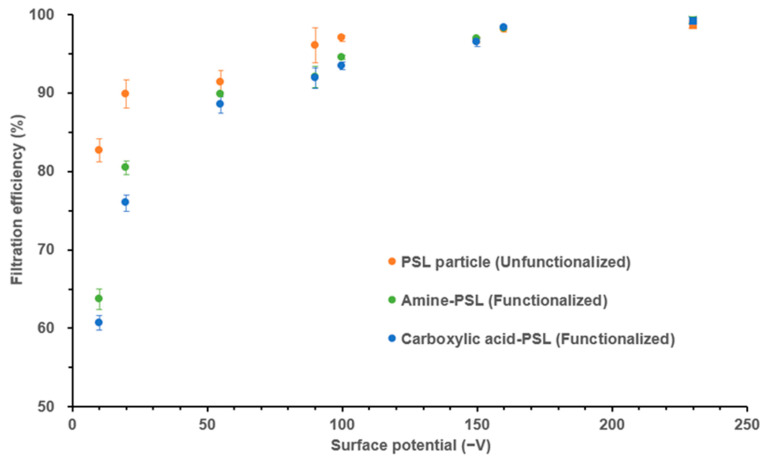
Comparisons of filtration efficiency for three different aerosolized particles (carboxylic acid-PSL, PSL, amine-PSL) among eight facial masks (one N95 mask shown as square shape and seven surgical masks shown as circle shape). The average mask filtration efficiency at 150 nm, obtained from triplicate experiments, is indicated by a colored dot. The error bars represent the standard deviation from the average of the triplicate experiments, and the different colors of the dots denote the use of different challenge particles. The test was performed under 40% RH.

The filter surface potential (Ψ_f_) varied significantly among the eleven mask samples analyzed in this study, ranging from −10 ± 5 V to −800 ± 108 V, with all masks carrying a negative Ψ_f_. Although there is no prescribed criterion for surface charge in N95 respirators, all N95-type masks are designed to use the electret filter to maintain high surface potential (net Ψ_f_ > 230 V in this study), which leads to a high filtration efficiency towards airborne particles (above 99% for 150 nm aerosolized PSL particles). In contrast, surgical masks do not always use a charged filter, resulting in a lower surface potential and potentially lower filtration efficiency for airborne particles. For instance, the surgical mask with low surface potential (Ψ_f_ = −10 ± 5 V) showed the poorest filtration efficiency (83%) (Table 1). Moreover, there was a clear trend of increasing filtration efficiency towards PSL particles among the surgical masks with increasing surface potential, as shown in Figure 4. In this study, we maintained the humidity level at 40% RH, as a higher humidity could weaken the static charge buildup ability, which results in a decrease in Ψ_f_ during the test [30,36]. The last section of this manuscript will consider humidity as the variable.

### 3.4. Impact of Aerosol Particle Surface Potential (Ψ_p_) on Filter Filtration Efficiency

Figure 4 illustrates the results of our experiment to assess the influence of particle surface charge on filtration efficiency. The 150 nm functionalized PSL particles (150 nm amine-PS or carboxylic acid-PS) with varying Ψ_p_ were used as the challenge aerosol on one N95 mask and seven surgical masks with different Ψ_f_. We observed that masks with higher surface charge had a higher filtration efficiency towards functionalized PSL particles, regardless of the charge on the particles. While a higher filtration efficiency with opposite charge seems obvious due to electrostatic interaction, an effect with the same charge was not expected and could potentially be the result of deflection into mask material. 

The N95 mask (Ψ_f_ = −230 ± 30 V) maintained a consistently higher filtration efficiency (> 98%) for either functionalized or unfunctionalized PSL challenge aerosols. However, surgical masks showed a range of efficiencies depending on the nature of particle charge, especially for those masks with low surface potential. The surgical mask with the lowest surface potential (Ψ_f_ = −10 ± 5 V) showed the highest filtration efficiency (83%) towards the 150 nm unfunctionalized PSL particles but a significantly lower filtration efficiency (~60%) towards the 150 nm functionalized PSL particles (carboxylic acid-PSL or amine-PSL). However, when looking at surgical masks with higher surface potential, like surface mask B (Ψ_f_ = −150 ± 20 V), the difference in filtration efficiency decreased and became not significant. Filtration efficiency stayed around 95%, no matter whether the challenge PSL aerosol was functionalized or unfunctionalized. The phenomenon observed may be explained by the presence of more highly charged particles for unfunctionalized PSL particles (Figure 3). These particles are more likely to be captured when the mask surface potential is low due to electrostatic interactions. This is consistent with a similar finding reported by Laengert et al. [52], who found that highly charged particles are more easily captured by filtration than minimally charged particles. Overall, PSL particle functionality significantly influences surface charge distribution, leading to variations in filtration efficiency when used as challenge materials to evaluate masks, especially those with low surface potential. 

Additionally, our study observed that amine-PSL (positively charged or neutral) particles showed slightly higher filtration efficiency compared to carboxylic acid-PSL (negatively charged) particles when tested with surgical masks F (*p* = 0.004) and G (*p* = 0.03), which have low surface potentials of Ψ_f_ = −20 ± 10 V and Ψ_f_ = −10 ± 5 V, respectively. This result could be caused by the electrostatic attraction between the negative mask surface potential and positive charges of the particles and suggests that an opposite polarity between mask surface charge and particle surface charge may increase the filtration efficiency. Moreover, this effect became insignificant as the mask surface potential increased. Further research is needed to fully understand the mechanism behind these observations.

### 3.5. Impact of Removing Mask Surface Charge on Filtration Efficiency

**Figure 5:**
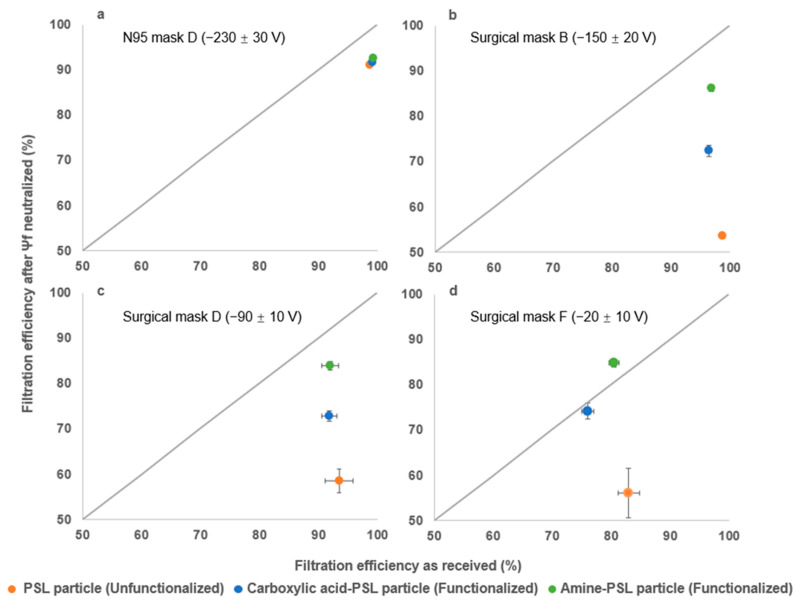
Comparison of the mask filtration efficiencies using three different aerosolized particles (carboxylic acid-PSL, PSL, and amine-PSL) for (**a**) N95 mask D, (**b**) surgical mask B, (**c**) surgical mask D and (**d**) surgical mask F before/after isopropanol treatment. The color-coded dot represents the mean filtration efficiency of triplicate experiments, while the error bar indicates the standard deviation from the mean value. Each color corresponds to a different type of challenge particle used in the experiment. The test was performed under 40% RH.

In the previous section, we studied the impact of the mask surface potential on filtration efficiency for differently charged particles. However, this was based on different mask products, so other production aspects could impact efficiency, too. In this section, we selected four different masks with varying Ψ_f_ and filtration efficiencies, including one N95 mask (D) and three surgical masks (B, D, F), to be treated by 2-propanol wash [57]. Each mask was then tested with the three different challenge aerosols as mentioned previously (150 nm carboxylic acid-PSL, PSL, amine-PSL); the representative testing results are shown in Figure 5.

Figure 5a shows that the filtration efficiency of the N95 mask decreased to around 90% from above 98% after surface charge neutralization, but it remained quite high consistently and independently of the challenge aerosol. In contrast to N95 masks, surgical masks exhibited varying filtration efficiency after surface charge neutralization that is dependent on the type of challenge aerosol. For instance, Figure 5b demonstrates that surgical mask B (with an original Ψ_f_ = −150 ± 20 V) had an original filtration efficiency over 95%, no matter the challenge aerosols. However, after neutralizing the surface charge, the filtration efficiency was reduced to 86%, 72%, and 54% for amine-PSL, carboxylic acid-PSL, and PSL particles, respectively. Similar trends were observed for surgical masks D and F, as shown in Figure 5c,d. Although there were differences in initial filtration efficiency and surface potential among surgical masks B, D, and F, their final filtration efficiencies for amine-PSL, carboxylic acid-PSL, and PSL particles remained consistent at approximately 85%, 70%, and 55%, respectively, following surface charge neutralization treatment. These results indicate that aerosolized particle surface charge may be a contributing factor to the observed differences. Moreover, an interesting phenomenon was observed where surgical mask F demonstrated a higher filtration efficiency (85%) after surface potential neutralization compared to its original value (80%). This is indicated by the dot above the 1:1 ratio in Figure 5d. To investigate why different PSL particles show varying filtration efficiency after mask surface charge neutralization, we measured the surface potential of the mask after each test to confirm the surface charge on the non-charged surface during the experiment. Even at the particle concentration used and rather short exposure times (10 min), we do see a static charge buildup on the filter surface. The post-experiment results showed the mask surface potential increased to Ψ_f_ = 40 ± 6 V and Ψ_f_ = −28 ± 4 V after challenge with amine-PSL particles (positively charged or neutral) and carboxylic acid-PSL particles (negatively charged), respectively. This would explain the observed higher filtration efficiency when surgical masks were challenged by amine-PSL particles compared to carboxylic acid-PSL particles due to the higher surface potential that originates from particle buildup and the surface potential now contributed by the surface charge on deposited particles (Figure 5b–d). In contrast, nebulized PSL particles caused the masks to maintain Ψ_f_ = 0 ± 3 V post-experiment. This outcome was due to the presence of both positively and negatively charged unfunctionalized PSL particles after the nebulization process [34,52], which led to surface charge neutralization while they built up on the non-charged mask surface and resulted in the lowest filtration efficiency (~55%). Although we lack SMPS spectrum data for positively charged particles, these results provide evidence that functionalized PSL particles tend to potentially carry monopolar charges, while PSL particles are bipolar charged after aerosolization. Additionally, functionalized PSL particles could alter the surface potential when deposited on a non-charged surface, in contrast to unfunctionalized PSL particles. However, the aerosol charge distribution after nebulization depends on several factors, including nebulization device, solution conductivity, temperature, suspension pH level, droplet size, and more [58,59], which are limited and hard to fully evaluate in a single study but would benefit from further investigation.

### 3.6. Impact of Humidity on Filtration Efficiency

As previously mentioned, humidity is known to have an impact on filtration efficiency, and the existing literature has reported the ability for static charge buildup to occur is weakened as humidity increases, especially over 40% RH [60,61,62]. However, masks are designed to be used in humid environments, as exhaled air from people typically has a humidity level over 90% [63]. Therefore, we evaluated the impact of humidity on our filtration efficiency tests by investigating a subset of eight different masks, including seven surgical masks and one N95 mask, challenging them with three differently charged model aerosols (carboxylic acid-PSL, PSL, and amine-PSL) at two different humidity levels (40% RH and 80% RH).

**Figure 6:**
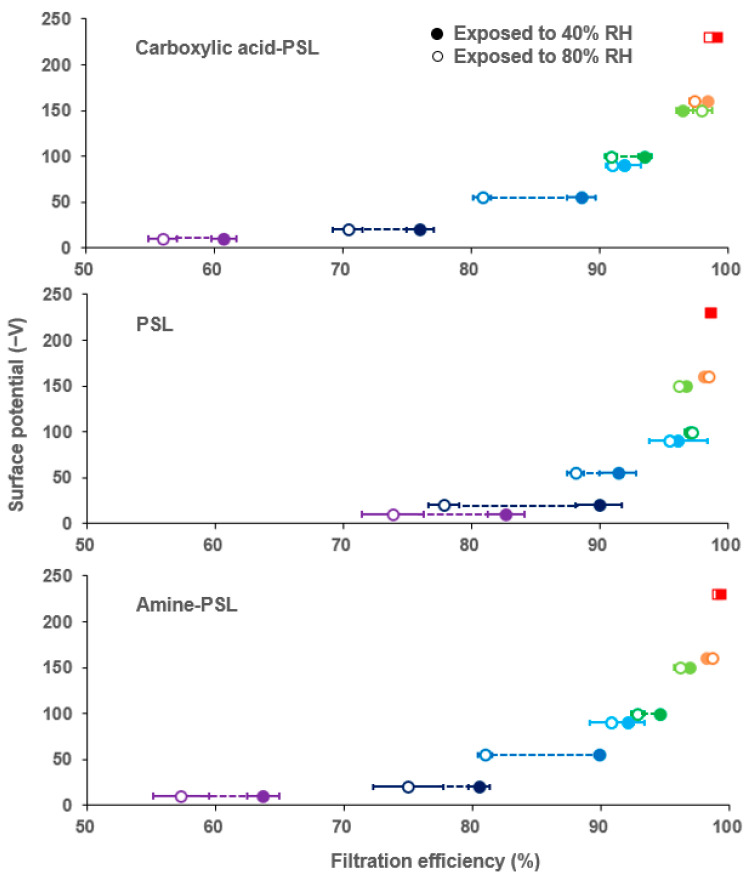
Filtration efficiency results for seven surgical masks (round) and one N95 mask (cubic) using three different particles (carboxylic acid-PSL, PSL, amine-PSL) at two humidity levels (40% RH, 80% RH). The filtration efficiency after the 15 min exposure test is represented by color-coded dots, where different colors represent the different mask samples, and the filling of each color represents the humidity level tested. The corresponding Ψ_f_ is shown on the y-axis, and the error bar indicates the standard deviation from the triplicate experiments. A dashed line was used to indicate significant differences in filtration efficiency for the sample tested at different humidity conditions.

According to the results shown in Figure 6, the N95 mask exhibited a consistent filtration efficiency (~98%) towards differently charged particles, independent of the humidity, in a short time exposure (15 min). In contrast, surgical masks exhibited a trend where masks that have lower Ψ_f_ are more affected by higher humidity environments, resulting in decreased filtration efficiency. Differently charged aerosols did not have a significant impact on filtration efficiency in this test. We measured the Ψ_f_ after each test. As the masks were slightly damp after these high humidity tests, static charge dissipated and led to Ψ_f_ around 0 ± 5 V for each test; this may be one of the reasons why the filtration efficiency decreased, as the electrostatic interaction was weakened, particularly for those masks that originally had low surface potential. In this test, both the challenge aerosols (PSL particles) are made of hydrophobic material, which ensures that the challenge aerosol remains within the target range (~150 nm) without significant growth (Figure S4). The mask material used in this experiment is also made of hydrophobic material, which prevents the mask from continuing to absorb moisture, resulting in a change in filtration efficiency [64].

Additionally, a 6 h prolonged test was conducted to investigate the long-term impact of humidity on mask surface potential. The Ψ_f_ of a surgical mask was measured after every 1 h exposure to air with 40% RH and 80% RH; measurements were recorded after the mask was completely dry to prevent readings from being affected by a wet surface. The results shown in Figure S5 indicate that the Ψ_f_ of the mask gradually decreased from −103 V to −18 V after the 6 h test. This suggests that a highly humid environment can significantly reduce the static charge of the mask, potentially resulting in decreases in filtration efficiency towards nanoscale aerosols.

## 4. Conclusions

This study investigated the effect of surface charge on mask materials and challenge aerosols on mask aerosol filtration efficiency. Monodisperse 150 nm functionalized and unfunctionalized PSL particles with varying surface charges were used to challenge eleven commercial masks, including N95 and surgical masks. The findings reveal that N95 masks consistently maintain a filtration efficiency exceeding 98%, regardless of changes in the surface charge of the challenge aerosol. In contrast, the filtration efficiency of surgical masks decreases as the surface potential of the mask decreases, ranging from 82% to 97% for corresponding surface potentials of −10 ± 5 V and −160 ± 18 V, respectively. Remarkably, functionalized PSL particles are captured less efficiently than unfunctionalized PSL particles, especially when the mask’s surface potential decreases, and the phenomenon is more observable. This finding needs to be further explored.

To eliminate the surface potential of both N95 and surgical masks, 2-propanol washes were employed, resulting in a surface potential of 0 ± 3 V after treatment. N95 masks exhibited a decrease in filtration efficiency to approximately 90%, regardless of the surface charge of the challenge aerosol. Similarly, surgical masks showed a decreasing trend in filtration efficiency. Notably, when functionalized PSL particles were conducted on a non-charged mask surface, the surface potential increased, leading to a higher filtration efficiency. Under higher humidity conditions (80% RH), the surface potential of each mask decreased to approximately 0 ± 5 V due to weakened surface charge buildup ability caused by moisture in the air. The filtration efficiency of the masks exhibited a decreasing trend compared to those tested under dry conditions (40% RH), especially when the mask surface potential was initially low. Furthermore, prolonged exposure to high humidity, such as that caused by exhalation, could accelerate the neutralization of mask surface charge and potentially decrease mask performance. 

This study highlights the critical role of electrostatic interactions in nanoscale aerosol capture, which is reflected in the surface potential of masks. Relative humidity conditions proved to also strongly impact filtration efficiency and charge distributions. The results provide important information on how the surface charge of both particles and masks affects mask performance.

## Figures and Tables

**Table 1:** Surface potential (Ψf), filtration efficiency, and pressure drop (∆P) of mask sample.

Mask Type	Sample Number	Surface Potential (Ψ_f_, V)	Filtration Efficiency (%)	Pressure Drop (mmH_2_O/cm^2^)
N95 mask	N95 mask A	−800 ± 108	99.2 ± 0.3	9
	N95 mask B	−539 ± 85	99.5 ± 0.2	7
	N95 mask C	−440 ± 53	99.1 ± 0.2	7
	N95 mask D	−230 ± 30	99.4 ± 0.2	6.5
Surgical mask	Surgical mask A	−160 ± 18	98.1 ± 0.3	8
	Surgical mask B	−150 ± 20	96.7 ± 0.4	5
	Surgical mask C	−100 ± 12	97.7 ± 0.4	4.5
	Surgical mask D	−90 ± 10	96.1 ± 2.3	7.5
	Surgical mask E	−55 ± 5	91.4 ± 1.4	3
	Surgical mask F	−20 ± 10	90.9 ± 1.8	4.5
	Surgical mask G	−10 ± 5	82.7 ± 1.4	4

Different letters (A, B, C, D, E, F, G) for N95 masks and surgical masks indicate the masks produced by different manufacturers.

## Data Availability

Data supporting the reported results will be provided upon reader’s request.

## Supplementary Materials

The following supporting information can be downloaded at: https://www.mdpi.com/article/10.3390/toxics12010003/s1, Figure S1: Muti-layer structure image of N95 mask and surgical mask, Figure S2: Comparison of mask filtration efficiencies with different flow directions, Figure S3: SEM images of each fiber layer for the masks used in this study, Figure S4: SMPS spectrum of particle size distribution while ambient humidity reaches 80% RH, Figure S5: Time-resolved decay of surface potential for surgical mask exposed to 40% RH and 80% RH air. Table S1: SMPS setting parameters, Table S2: Zeta potential and particle size distribution for each challenge aerosol at nebulized pH level, Table S3: Remeasured surface potential of masks used for filtration efficiency test, after 2 years of storage, Table S4: Quality factor of the masks tested, Table S5: Number of charges on particle and corresponding electrical mobility diameter.
