# Supplementary material for: Do Surface Charges on Polymeric Filters and Airborne Particles Control the Removal of Nanoscale Aerosols by Polymeric Facial Masks?
Source: Toxics. 2023 Dec 19;12(1):3. doi: 10.3390/toxics12010003 (PMC10821015; doi:10.3390/toxics12010003)
Supplement: Supplementary file 1 [file toxics-12-00003-s001.zip › toxics-2718148-supplementary.pdf]

toxics

Supplementary materials

Do Surface Charges on Polymeric Filters and Airborne Particles Control the Removal of Nanoscale Aerosols by Polymeric Facial Masks?

Zhaobo Zhang [1], Mahmut S. Ersan [2,3], Paul Westerhoff [2] and Pierre Herckes [1,*]

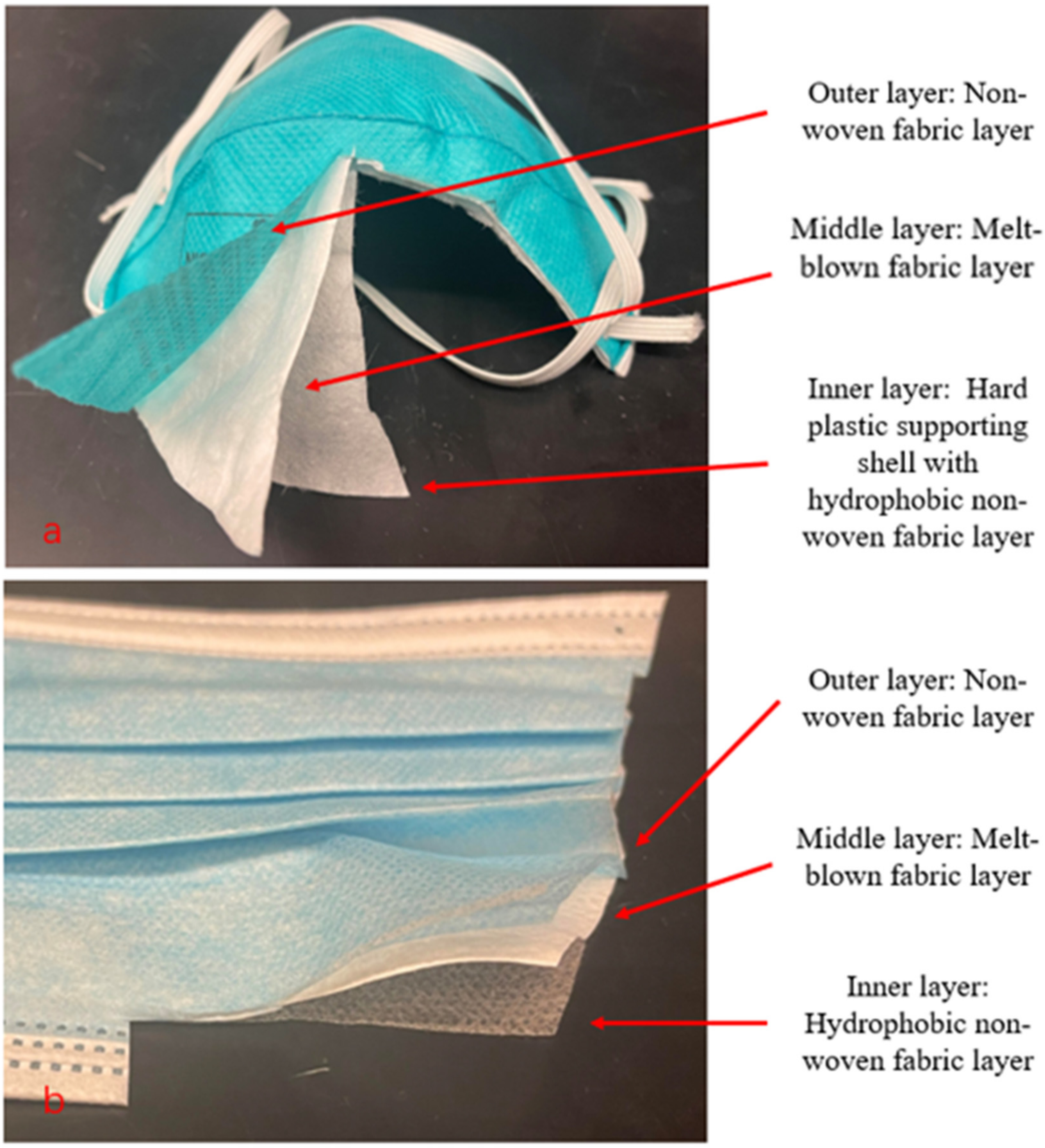

Figure S1. Muti-layer structure image of (a) N95 mask and (b) surgical mask.

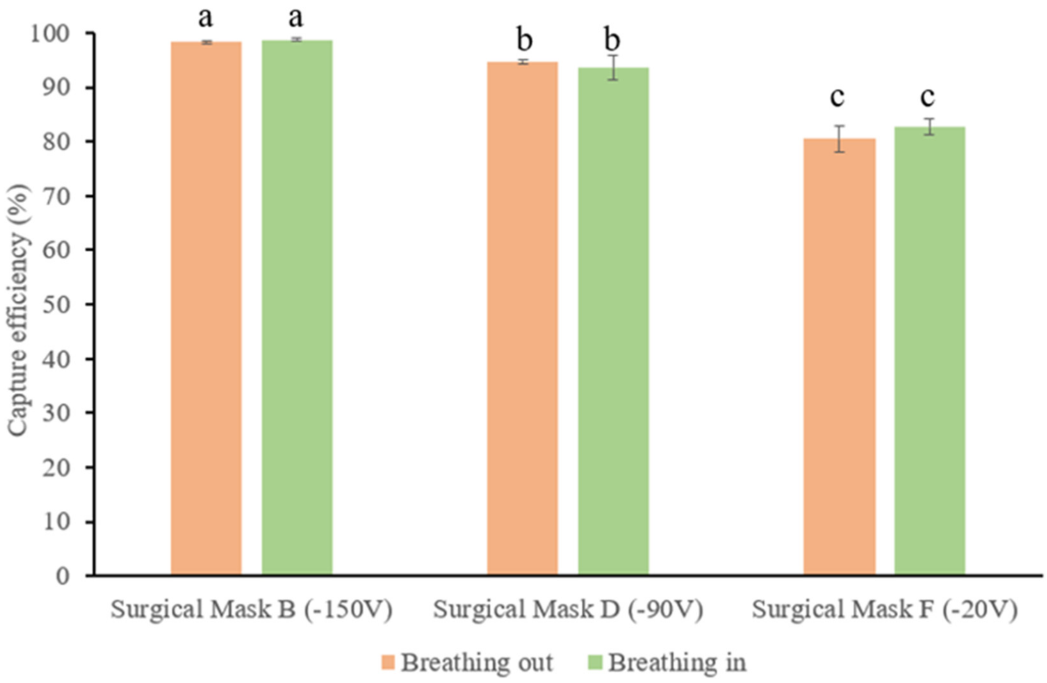

Figure S2. Comparison of mask filtration efficiencies with different flow directions, which represent the capture efficiency for breathing in and breathing out. Different letters (a, b, c) on the colored bars mean the experimental results are statistically different ($p < 0.05$) based on a two-tailed unpaired Student's t-test.

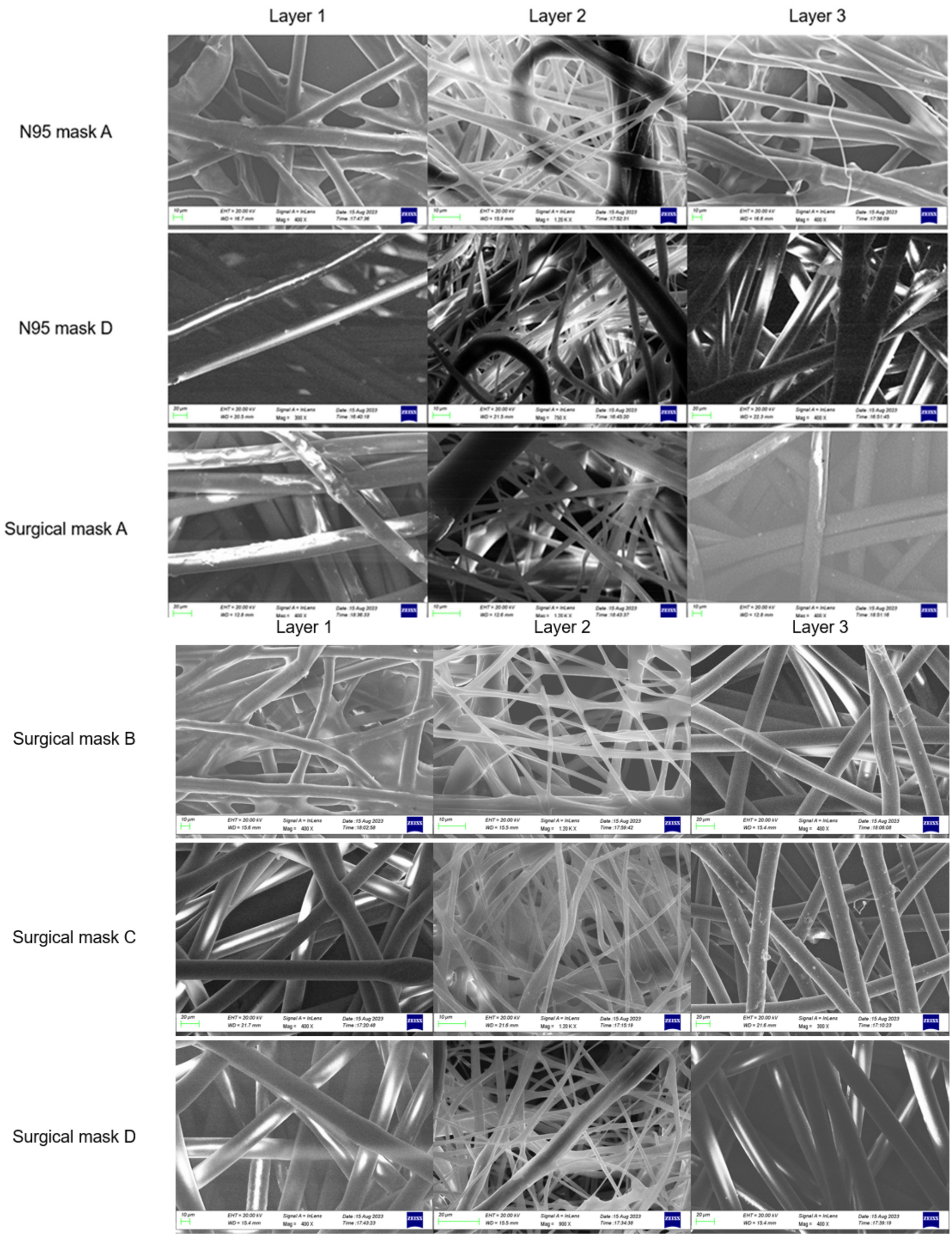
Layer 1
Layer 2
Layer 3
N95 mask A
N95 mask D
Surgical mask A
Layer 1
Layer 2
Layer 3
Surgical mask B
Surgical mask C
Surgical mask D

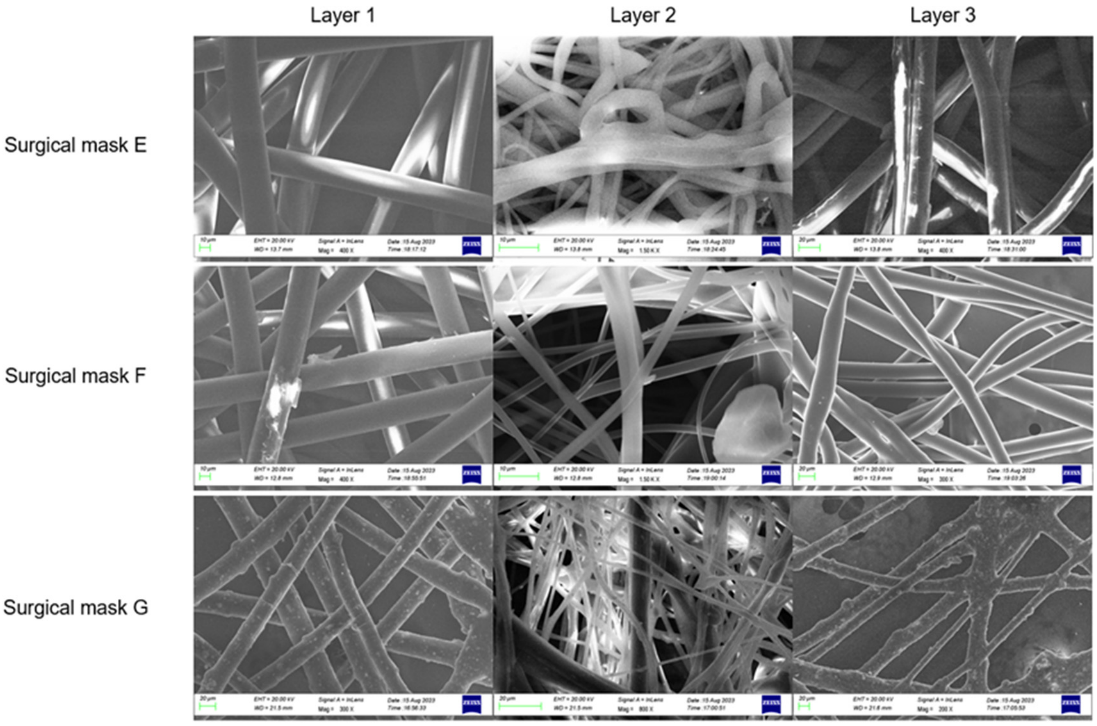

Figure S3. SEM images of each fiber layer for the masks used in this study.

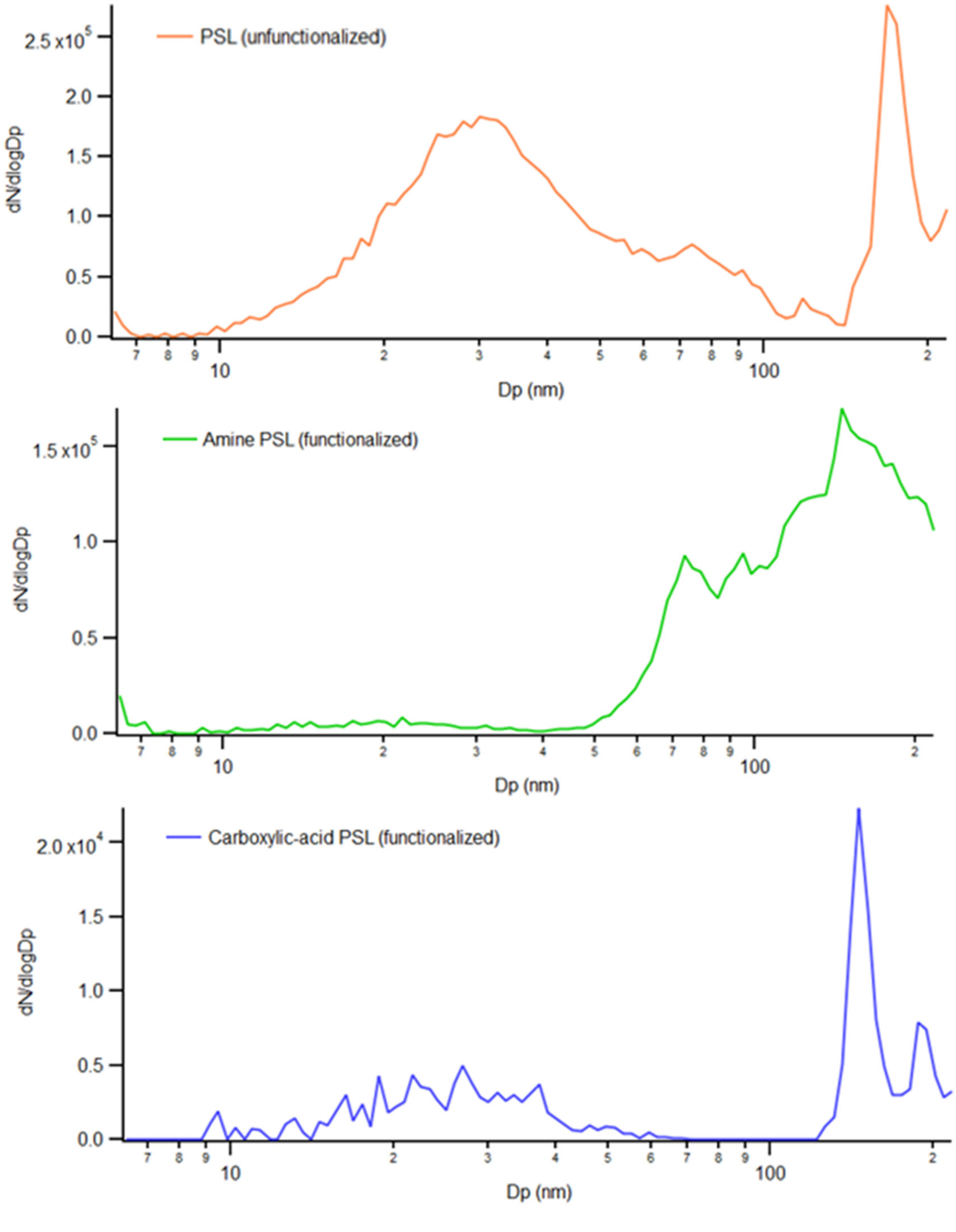

Figure S4. SMPS spectrum of particle (150 nm PSL, amine-PSL, and carboxylic acid-PSL) size distribution while ambient humidity reaches 80% RH. The maximum peak is still around 150 nm without significant growth.

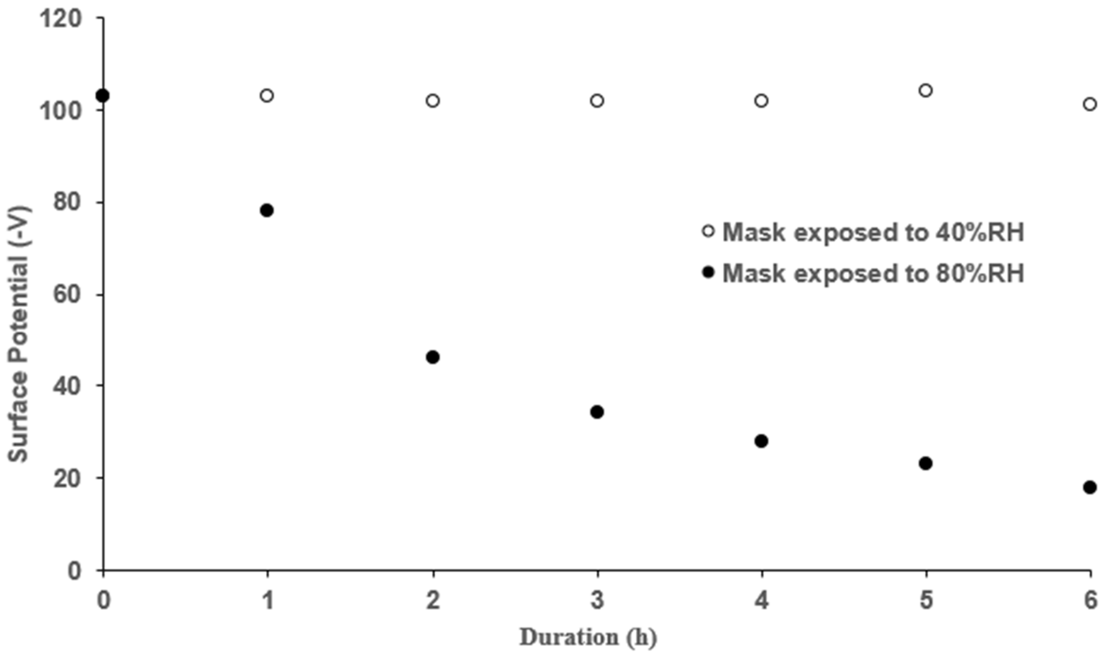

Figure S5. Time-resolved decay of surface potential for surgical mask B exposed to 40% RH and 80% RH air.

Table S1. SMPS setting parameters.

Reference gas viscosity (Pa × s)	1.83×10^{-5}
Gas temperature (K)	297.15
Reference gas temperature (K)	296.15
Gas pressure (kPa)	95
Reference gas pressure (kPa)	101.3
Reference gas mean free path (m)	6.73×10^{-8}

Table S2. Zeta potential and size distribution for each challenge aerosol at nebulized pH .

Challenge aerosol	Zeta potential (mV)	Mean Diameter (nm) N=5	pH of nebulization fluid
Carboxylic-acid PSL	-60 ± 3	150 ± 2	6.06
PSL	-22 ± 2	150 ± 4	6.23
Amine-PSL	21 ± 1	150 ± 6	9.12

Note: Mean diameter measured by Dynamic light scattering (DLS).

Table S3. Remeasured surface potential of masks used for filtration efficiency test, after 2 years of storage.

Mask type	Sample number	Surface potential for test (V)	Remeasured surface potential after two years (V)
N95 mask	N95 mask A	-800 ± 108	-765 ± 144
	N95 mask B	-539 ± 85	Material ran out
	N95 mask C	-440 ± 53	Material ran out
	N95 mask D	-230 ± 30	-253 ± 48
Surgical mask	Surgical mask A	-160 ± 18	-166 ± 28
	Surgical mask B	-150 ± 20	-124 ± 19
	Surgical mask C	-100 ± 12	-40± 5
	Surgical mask D	- 90 ± 10	-103 ± 26
	Surgical mask E	-55 ± 5	-66 ± 12
	Surgical mask F	-20 ± 10	-18 ± 6
	Surgical mask G	-10 ± 5	-13 ± 4

Note: Only surgical mask C shows significant surface charge dissipation after 2 years of storage.

$$Quality\ Factor = \frac{-\ln\left(1 - \frac{FE_{min}}{100}\right)}{\Delta \mathrm{P}}$$

Table S4. Quality factor of the masks tested.

Mask type	Sample number	Quality Factor (kPa^{-1})
N95 mask	N95 mask A	70.6
	N95 mask B	77.2
	N95 mask C	68.7
	N95 mask D	80.3
Surgical mask	Surgical mask A	50.5
	Surgical mask B	69.6
	Surgical mask C	85.7
	Surgical mask D	44.1
	Surgical mask E	83.4
	Surgical mask F	54.5
	Surgical mask G	43.5

Table S5. Number of charges on particle and corresponding electrical mobility diameter.

Number of charges	Amine-PSL	PSL	Carboxylic acid-PSL
	Electrical Mobility Diameter (nm)		
1	151	168	146
2	100	111	97
3	80	88	77
4	68	75	66
5	60	66	59
6	55	60	53
7	50	55	49
8	47	51	46
9	44	48	43
10	42	46	41
20	29	32	28
30	24	26	23
40	20	22	20
50	18	20	18
60	16	18	16
70	15	17	15
80	14	16	14
90	13	15	13
100	13	14	12
200	8	9	8
300	6	7	6
